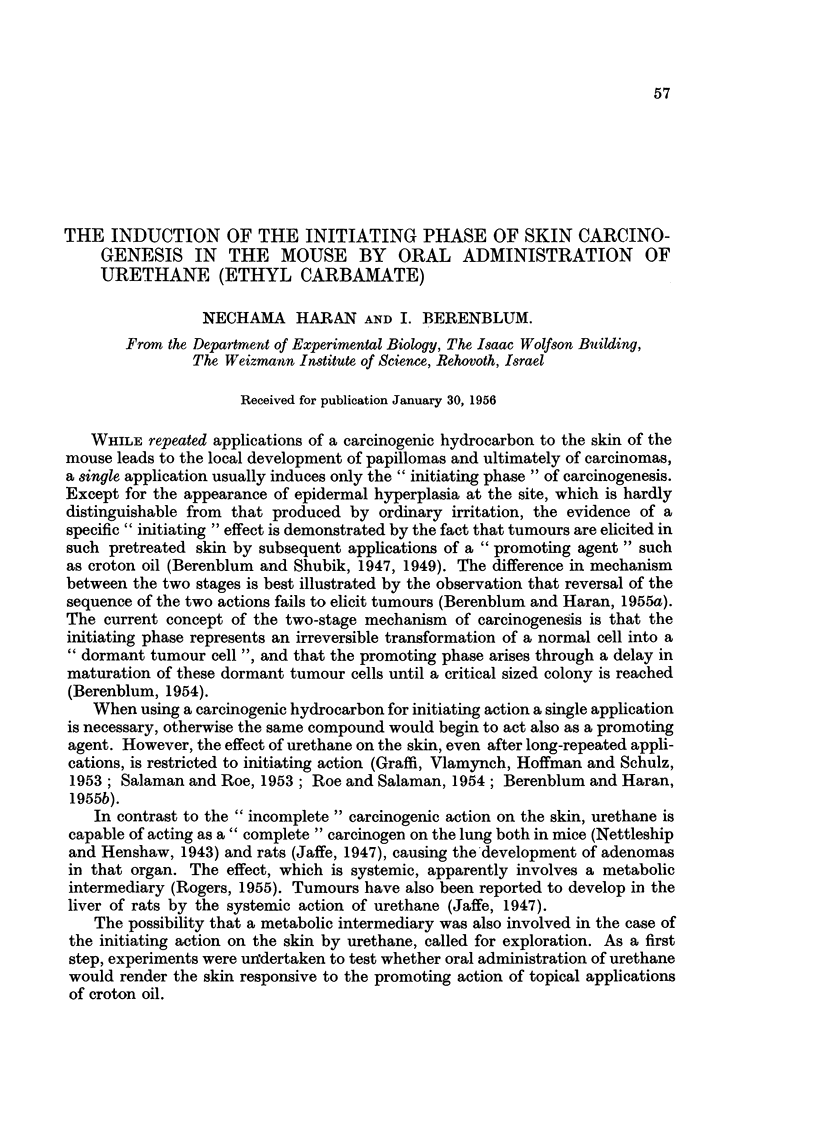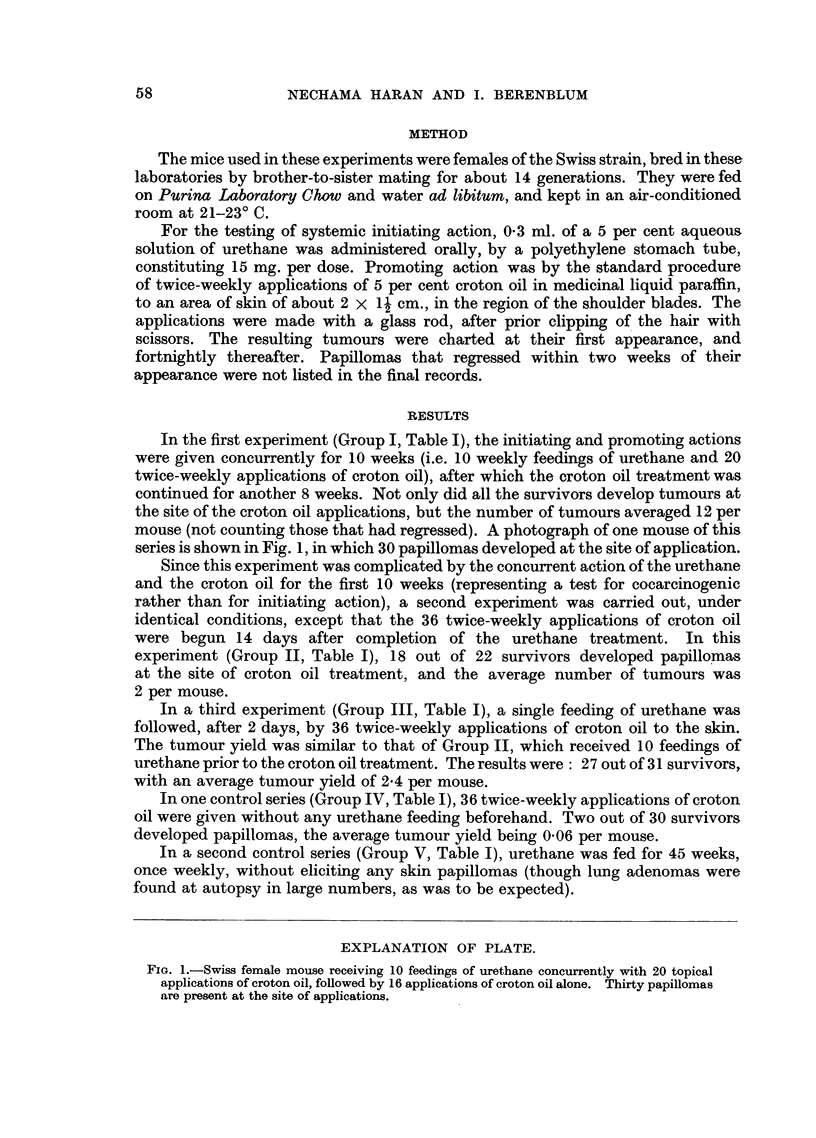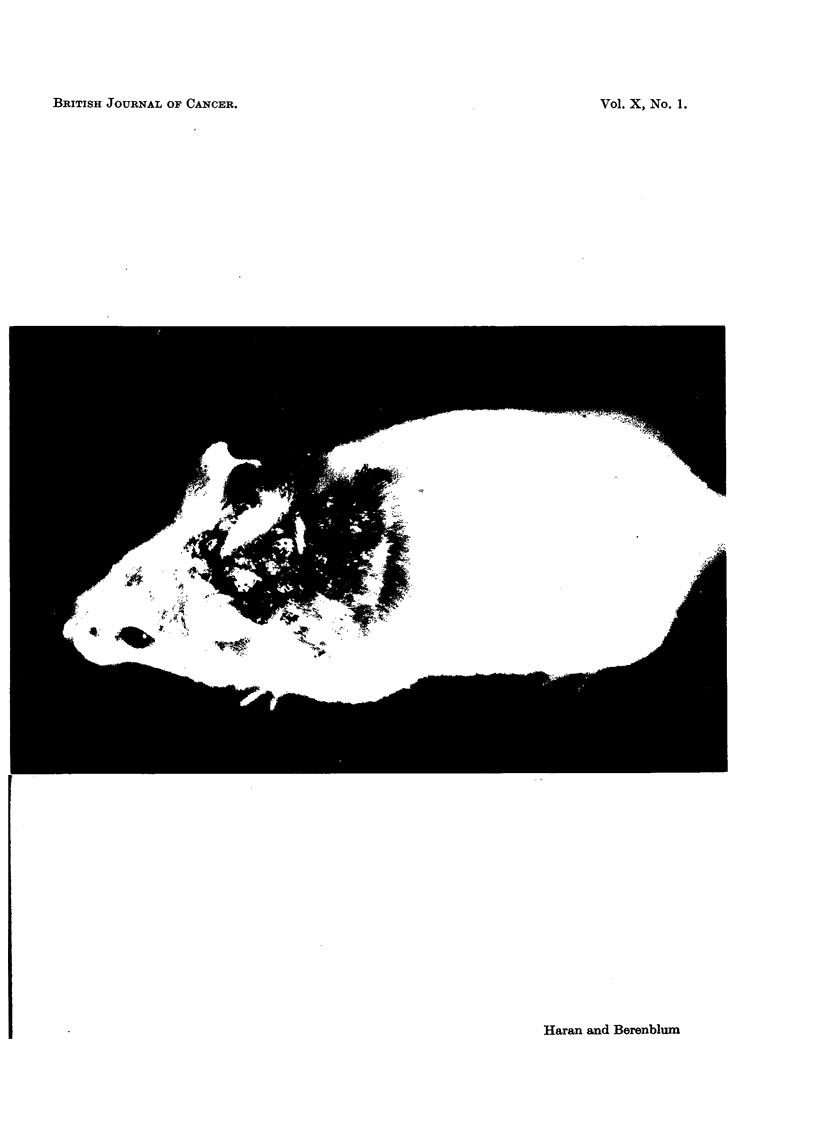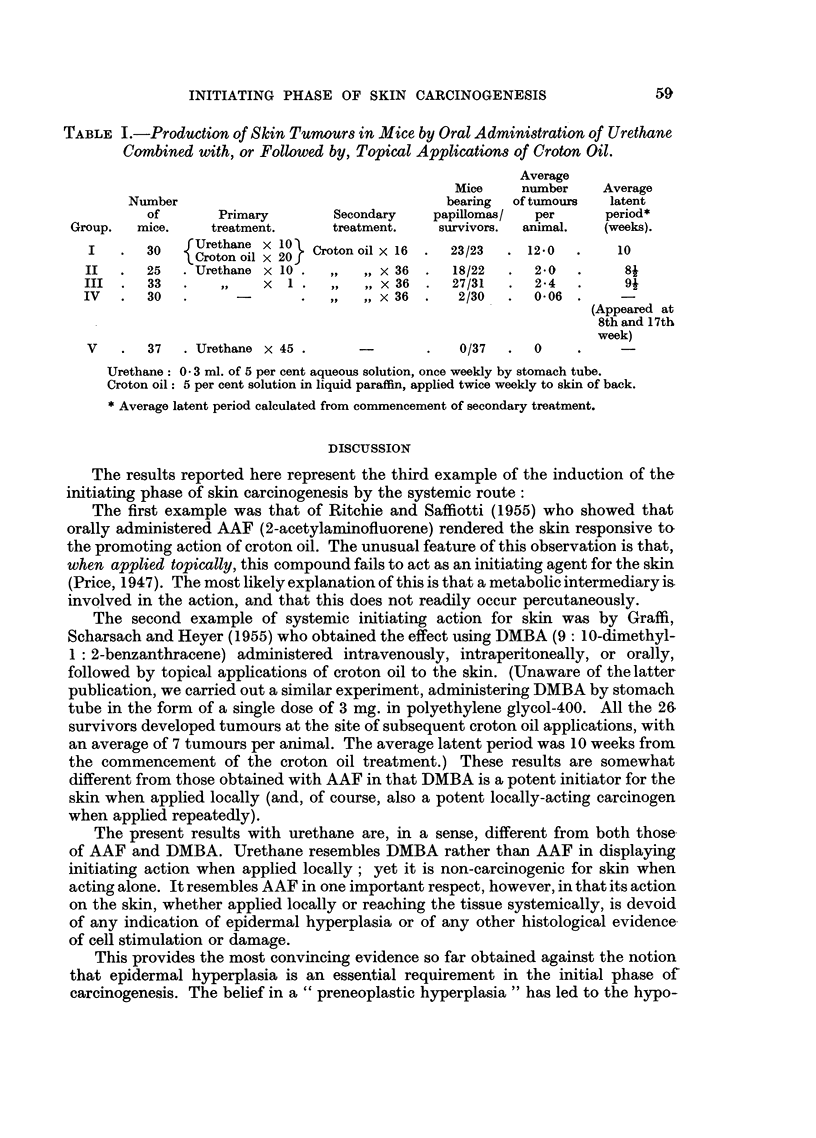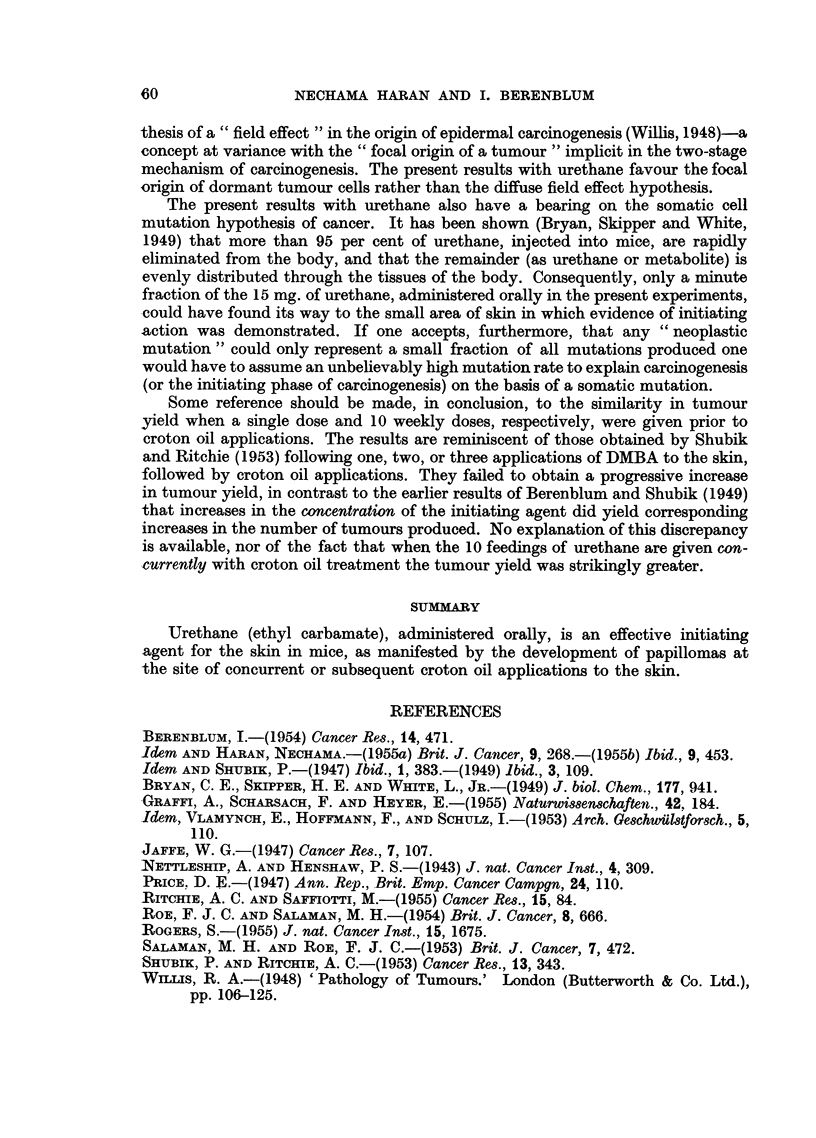# The Induction of the Initiating Phase of Skin Carcinogenesis in the Mouse by Oral Administration of Urethane (Ethyl Carbamate)

**DOI:** 10.1038/bjc.1956.8

**Published:** 1956-03

**Authors:** Nechama Haran, I. Berenblum

## Abstract

**Images:**


### THE INDUCTION OF THE INITIATING PHASE OF SKIN CARCINOGENESIS IN THE MOUSE BY ORAL ADMINISTRATION OF URETHANE (ETHYL CARBAMATE)

NECHAMA HARAN AND I. BERENBLUM.

*From the Department of Experimental Biology, The Isaac Wolfson Building, The Weizmann Institute of Science, Rehovoth, Israel*

Received for publication January 30, 1956

WHILE *repeated* applications of a carcinogenic hydrocarbon to the skin of the mouse leads to the local development of papillomas and ultimately of carcinomas, a *single* application usually induces only the " initiating phase " of carcinogenesis. Except for the appearance of epidermal hyperplasia at the site, which is hardly distinguishable from that produced by ordinary irritation, the evidence of a specific " initiating " effect is demonstrated by the fact that tumours are elicited in such pretreated skin by subsequent applications of a " promoting agent " such as croton oil (Berenblum and Shubik, 1947, 1949). The difference in mechanism between the two stages is best illustrated by the observation that reversal of the sequence of the two actions fails to elicit tumours (Berenblum and Haran, 1955*a*). The current concept of the two-stage mechanism of carcinogenesis is that the initiating phase represents an irreversible transformation of a normal cell into a " dormant tumour cell ", and that the promoting phase arises through a delay in maturation of these dormant tumour cells until a critical sized colony is reached (Berenblum, 1954).

When using a carcinogenic hydrocarbon for initiating action a single application is necessary, otherwise the same compound would begin to act also as a promoting agent. However, the effect of urethane on the skin, even after long-repeated applications, is restricted to initiating action (Graffi, Vlamynch, Hoffman and Schulz, 1953 ; Salaman and Roe, 1953 ; Roe and Salaman, 1954 ; Berenblum and Haran, 1955*b*).

In contrast to the " incomplete " carcinogenic action on the skin, urethane is capable of acting as a " complete " carcinogen on the lung both in mice (Nettleship and Henshaw, 1943) and rats (Jaffe, 1947), causing the development of adenomas in that organ. The effect, which is systemic, apparently involves a metabolic intermediary (Rogers, 1955). Tumours have also been reported to develop in the liver of rats by the systemic action of urethane (Jaffe, 1947).

The possibility that a metabolic intermediary was also involved in the case of the initiating action on the skin by urethane, called for exploration. As a first step, experiments were undertaken to test whether oral administration of urethane would render the skin responsive to the promoting action of topical applications of croton oil.

### METHOD

The mice used in these experiments were females of the Swiss strain, bred in these laboratories by brother-to-sister mating for about 14 generations. They were fed on *Purina Laboratory Chow* and water *ad libitum*, and kept in an air-conditioned room at 21–23° C.

For the testing of systemic initiating action, 0·3 ml. of a 5 per cent aqueous solution of urethane was administered orally, by a polyethylene stomach tube, constituting 15 mg. per dose. Promoting action was by the standard procedure of twice-weekly applications of 5 per cent croton oil in medicinal liquid paraffin, to an area of skin of about 2 × $1\frac{1}{2}$ cm., in the region of the shoulder blades. The applications were made with a glass rod, after prior clipping of the hair with scissors. The resulting tumours were charted at their first appearance, and fortnightly thereafter. Papillomas that regressed within two weeks of their appearance were not listed in the final records.

### RESULTS

In the first experiment (Group I, Table I), the initiating and promoting actions were given concurrently for 10 weeks (i.e. 10 weekly feedings of urethane and 20 twice-weekly applications of croton oil), after which the croton oil treatment was continued for another 8 weeks. Not only did all the survivors develop tumours at the site of the croton oil applications, but the number of tumours averaged 12 per mouse (not counting those that had regressed). A photograph of one mouse of this series is shown in Fig. 1, in which 30 papillomas developed at the site of application.

Since this experiment was complicated by the concurrent action of the urethane and the croton oil for the first 10 weeks (representing a test for cocarcinogenic rather than for initiating action), a second experiment was carried out, under identical conditions, except that the 36 twice-weekly applications of croton oil were begun 14 days after completion of the urethane treatment. In this experiment (Group II, Table I), 18 out of 22 survivors developed papillomas at the site of croton oil treatment, and the average number of tumours was 2 per mouse.

In a third experiment (Group III, Table I), a single feeding of urethane was followed, after 2 days, by 36 twice-weekly applications of croton oil to the skin. The tumour yield was similar to that of Group II, which received 10 feedings of urethane prior to the croton oil treatment. The results were : 27 out of 31 survivors, with an average tumour yield of 2·4 per mouse.

In one control series (Group IV, Table I), 36 twice-weekly applications of croton oil were given without any urethane feeding beforehand. Two out of 30 survivors developed papillomas, the average tumour yield being 0·06 per mouse.

In a second control series (Group V, Table I), urethane was fed for 45 weeks, once weekly, without eliciting any skin papillomas (though lung adenomas were found at autopsy in large numbers, as was to be expected).

---

EXPLANATION OF PLATE.

FIG. 1.—Swiss female mouse receiving 10 feedings of urethane concurrently with 20 topical applications of croton oil, followed by 16 applications of croton oil alone. Thirty papillomas are present at the site of applications.

Haran and Berenblum

TABLE I.—*Production of Skin Tumours in Mice by Oral Administration of Urethane Combined with, or Followed by, Topical Applications of Croton Oil.*

| Group. | Number of mice. | Primary treatment. | Secondary treatment. | Mice bearing papillomas/ survivors. | Average number of tumours per animal. | Average latent period* (weeks). |
|---|---|---|---|---|---|---|
| I | 30 | {Urethane × 10, Croton oil × 20} | Croton oil × 16 | 23/23 | 12·0 | 10 |
| II | 25 | Urethane × 10 | „ „ × 36 | 18/22 | 2·0 | 8½ |
| III | 33 | „ × 1 | „ „ × 36 | 27/31 | 2·4 | 9½ |
| IV | 30 | — | „ „ × 36 | 2/30 | 0·06 | — (Appeared at 8th and 17th week) |
| V | 37 | Urethane × 45 | — | 0/37 | 0 | — |

Urethane : 0·3 ml. of 5 per cent aqueous solution, once weekly by stomach tube.
Croton oil : 5 per cent solution in liquid paraffin, applied twice weekly to skin of back.

* Average latent period calculated from commencement of secondary treatment.

### DISCUSSION

The results reported here represent the third example of the induction of the initiating phase of skin carcinogenesis by the systemic route :

The first example was that of Ritchie and Saffiotti (1955) who showed that orally administered AAF (2-acetylaminofluorene) rendered the skin responsive to the promoting action of croton oil. The unusual feature of this observation is that, *when applied topically*, this compound fails to act as an initiating agent for the skin (Price, 1947). The most likely explanation of this is that a metabolic intermediary is involved in the action, and that this does not readily occur percutaneously.

The second example of systemic initiating action for skin was by Graffi, Scharsach and Heyer (1955) who obtained the effect using DMBA (9 : 10-dimethyl-1 : 2-benzanthracene) administered intravenously, intraperitoneally, or orally, followed by topical applications of croton oil to the skin. (Unaware of the latter publication, we carried out a similar experiment, administering DMBA by stomach tube in the form of a single dose of 3 mg. in polyethylene glycol-400. All the 26 survivors developed tumours at the site of subsequent croton oil applications, with an average of 7 tumours per animal. The average latent period was 10 weeks from the commencement of the croton oil treatment.) These results are somewhat different from those obtained with AAF in that DMBA is a potent initiator for the skin when applied locally (and, of course, also a potent locally-acting carcinogen when applied repeatedly).

The present results with urethane are, in a sense, different from both those of AAF and DMBA. Urethane resembles DMBA rather than AAF in displaying initiating action when applied locally ; yet it is non-carcinogenic for skin when acting alone. It resembles AAF in one important respect, however, in that its action on the skin, whether applied locally or reaching the tissue systemically, is devoid of any indication of epidermal hyperplasia or of any other histological evidence of cell stimulation or damage.

This provides the most convincing evidence so far obtained against the notion that epidermal hyperplasia is an essential requirement in the initial phase of carcinogenesis. The belief in a " preneoplastic hyperplasia " has led to the hypothesis of a " field effect " in the origin of epidermal carcinogenesis (Willis, 1948)—a concept at variance with the " focal origin of a tumour " implicit in the two-stage mechanism of carcinogenesis. The present results with urethane favour the focal origin of dormant tumour cells rather than the diffuse field effect hypothesis.

The present results with urethane also have a bearing on the somatic cell mutation hypothesis of cancer. It has been shown (Bryan, Skipper and White, 1949) that more than 95 per cent of urethane, injected into mice, are rapidly eliminated from the body, and that the remainder (as urethane or metabolite) is evenly distributed through the tissues of the body. Consequently, only a minute fraction of the 15 mg. of urethane, administered orally in the present experiments, could have found its way to the small area of skin in which evidence of initiating action was demonstrated. If one accepts, furthermore, that any " neoplastic mutation " could only represent a small fraction of all mutations produced one would have to assume an unbelievably high mutation rate to explain carcinogenesis (or the initiating phase of carcinogenesis) on the basis of a somatic mutation.

Some reference should be made, in conclusion, to the similarity in tumour yield when a single dose and 10 weekly doses, respectively, were given prior to croton oil applications. The results are reminiscent of those obtained by Shubik and Ritchie (1953) following one, two, or three applications of DMBA to the skin, followed by croton oil applications. They failed to obtain a progressive increase in tumour yield, in contrast to the earlier results of Berenblum and Shubik (1949) that increases in the *concentration* of the initiating agent did yield corresponding increases in the number of tumours produced. No explanation of this discrepancy is available, nor of the fact that when the 10 feedings of urethane are given *concurrently* with croton oil treatment the tumour yield was strikingly greater.

### SUMMARY

Urethane (ethyl carbamate), administered orally, is an effective initiating agent for the skin in mice, as manifested by the development of papillomas at the site of concurrent or subsequent croton oil applications to the skin.

### REFERENCES

Berenblum, I.—(1954) *Cancer Res.*, **14**, 471.
*Idem* and Haran, Nechama.—(1955*a*) *Brit. J. Cancer*, **9**, 268.—(1955*b*) *Ibid.*, **9**, 453.
*Idem* and Shubik, P.—(1947) *Ibid.*, **1**, 383.—(1949) *Ibid.*, **3**, 109.
Bryan, C. E., Skipper, H. E. and White, L., Jr.—(1949) *J. biol. Chem.*, **177**, 941.
Graffi, A., Scharsach, F. and Heyer, E.—(1955) *Naturwissenschaften.*, **42**, 184.
*Idem*, Vlamynch, E., Hoffmann, F., and Schulz, I.—(1953) *Arch. Geschwülstforsch.*, **5**, 110.
Jaffe, W. G.—(1947) *Cancer Res.*, **7**, 107.
Nettleship, A. and Henshaw, P. S.—(1943) *J. nat. Cancer Inst.*, **4**, 309.
Price, D. E.—(1947) *Ann. Rep.*, *Brit. Emp. Cancer Campgn*, **24**, 110.
Ritchie, A. C. and Saffiotti, M.—(1955) *Cancer Res.*, **15**, 84.
Roe, F. J. C. and Salaman, M. H.—(1954) *Brit. J. Cancer*, **8**, 666.
Rogers, S.—(1955) *J. nat. Cancer Inst.*, **15**, 1675.
Salaman, M. H. and Roe, F. J. C.—(1953) *Brit. J. Cancer*, **7**, 472.
Shubik, P. and Ritchie, A. C.—(1953) *Cancer Res.*, **13**, 343.
Willis, R. A.—(1948) 'Pathology of Tumours.' London (Butterworth & Co. Ltd.), pp. 106–125.